# Intestinal lysozyme engagement of *Salmonella Typhimurium* stimulates the release of barrier-impairing InvE and Lpp1

**DOI:** 10.1016/j.jbc.2024.107424

**Published:** 2024-05-31

**Authors:** Jiangmeng Han, Iyshwarya Balasubramanian, Juan A. Flores, Sheila Bandyopadhyay, Jiaxing Yang, Yue Liu, Rajbir Singh, Prashanth Setty, Pawel Kiela, Ronaldo Ferraris, Nan Gao

**Affiliations:** 1 Department of Biological Sciences, Rutgers University, Newark, New Jersey, USA; 2 Department of Pediatrics, Daniel Cracchiolo Institute for Pediatric Autoimmune Disease Research, Steele Children's Research Center, University of Arizona, Tucson, Arizona, USA; 3 Department of Pharmacology, Physiology, and Neuroscience, Rutgers Biomedical and Health Sciences, Newark, New Jersey, USA

**Keywords:** lysozyme, *Salmonella Typhimurium*, virulence, infection, intestine, barrier function, Lpp 1, sipC, InvE

## Abstract

Lysozyme is a β-1,4-glycosidase that hydrolyzes the polysaccharide backbone of bacterial cell walls. With an additional bactericidal function mediated by a separate protein domain, lysozyme is considered a uniquely important antimicrobial molecule contributing to the host's innate immune response to infection. Elevated lysozyme production is found in various inflammatory conditions while patients with genetic risks for inflammatory bowel diseases demonstrate abnormal lysozyme expression, granule packaging, and secretion in Paneth cells. However, it remains unclear how a gain- or loss-of-function in host lysozyme may impact the host inflammatory responses to pathogenic infection. We challenged *Lyz1*^*−/−*^ and ectopic *Lyz1*-expressing (*Villin-Lyz1*^*TG*^) mice with *S*. *Typhimurium* and then comprehensively assessed the inflammatory disease progression. We conducted proteomics analysis to identify molecules derived from human lysozyme-mediated processing of live *Salmonella*. We examined the barrier-impairing effects of these identified molecules in human intestinal epithelial cell monolayer and enteroids. *Lyz1*^*−/−*^ mice are protected from infection in terms of morbidity, mortality, and barrier integrity, whereas *Villin-Lyz1*^*TG*^ mice demonstrate exacerbated infection and inflammation. The growth and invasion of *Salmonella in vitro* are not affected by human or chicken lysozyme, whereas lysozyme encountering of live *Salmonella* stimulates the release of barrier-disrupting factors, InvE-sipC and Lpp1, which directly or indirectly impair the tight junctions. The direct engagement of host intestinal lysozyme with an enteric pathogen such as *Salmonella* promotes the release of virulence factors that are barrier-impairing and pro-inflammatory. Controlling lysozyme function may help alleviate the inflammatory progression.

Lysozyme, also known as muramidase, is a 14-kDa enzyme found in various mucosal tissues and secretions. Through catalytic cleavage of the β-1,4-glycosidic bond between the N-acetylmuramic acid and N-acetylglucosamide that form the peptidoglycan monomers, lysozyme promotes the hydrolysis of polysaccharide backbone of the bacterial cell wall. As a part of the innate immune system, lysozymes are categorized into the c-type (or chicken type), the g-type (goose type), and the i-type (invertebrate type) (1). The lysozyme in chicken egg white was the first extensively studied lysozyme at biochemical and functional levels. Along with the human lysozyme, both are c-type isoforms and share approximately 59% sequence identity. Biochemical analysis of chicken lysozyme revealed a helix-loop-helix bactericidal domain mediating its membrane-permeabilizing action (2, 3). The human lysozyme exhibits a higher antibacterial activity than the chicken isoform, and intriguingly, the bactericidal activity of lysozyme is structurally and functionally separate from its catalytic domain (4). Amino acids responsible for lysozyme’s enzymatic and bactericidal functions are evolutionarily conserved across animal species. These dual mechanistic actions of lysozymes uniquely distinguish them from the other antimicrobial peptides, as the majority of the latter do not possess enzymatic functions.

The human lysozyme is encoded by the *LYZ* gene on chromosome 12q15 and can be abundantly produced by Paneth cells that reside in the small intestinal epithelia as well as by innate immune cells such as macrophages and neutrophils. Mice have two lysozyme genes, *Lyz1* and *Lyz2*, contributing to the lysozyme proteins predominantly found in the Paneth cells and the macrophages, respectively. Paneth cells are crypt-localized intestinal epithelial cells secreting lysozyme into the gut lumen where the commensal microbiota colonize (5). By genetically disrupting the *Lyz1* gene in mice, we have previously reported that the basal Nod-like receptor microbial signaling pathway is weakened and the commensal bacterial landscape is altered (6). In *Lyz1*^*−/−*^ mouse intestines, there are increased goblet and tuft cell populations, which are caused by an elevated basal type 2 immune response originating from the innate lymphocyte 2 in the lamina propria (6). Loss or gain of function in intestinal lysozyme in mice dampened or exacerbated dextran sulfate sodium-induced colitis, suggesting that proper control of the abundance of intestinal luminal lysozyme is important for inflammatory disease progression (6).

Elevated fecal lysozyme has been reported in patients with inflammatory bowel diseases (IBD) (7, 8, 9, 10, 11, 12). Interestingly, elevated lysozyme was observed in the colonic epithelia containing metaplastic Paneth-like cells in ulcerative colitis patients (13). Patients with IBD carrying *ATG16L1 T300A*, or *NOD2* disease-associated alleles show abnormal lysozyme expression and packaging in their Paneth cells (14, 15). Mutation in a major IBD risk gene LRRK2 was shown to disrupt lysozyme packaging in the dense core secretory granules in Paneth cells (16). Given the reported bactericidal activity, these reports suggest a potential host response to diminish the adherence or colonization by opportunistic pathobiont or pathogenic bacteria at the intestinal epithelial surface.

In terms of lysozyme interaction with pathogenic bacteria, it was reported in mice that *S*. *Typhimurium* infection reduces lysozyme production in Paneth cells (17), and the infection stimulates Paneth cells to release lysozyme *via* a secretory autophagy pathway (5). However, it was unclear if a gain or loss of function in host lysozyme may impact the host–pathogen interaction thereby affecting the pathogenesis and inflammatory responses caused by infection. In this report, we performed *in vivo S. Typhimurium* infection studies and comprehensively assessed the inflammatory disease progression in genetically modified *Lyz1*^*−/−*^ and *Lyz1*-overexpressing (*Villin-Lyz1*^*TG*^) mice. We found that increased intestinal lysozyme production exacerbates the morbidity and mortality following infection while diminishing intestinal lysozyme unexpectedly dampens the inflammatory progression triggered by the infection. *In vivo*, barrier function analysis demonstrated that mice with an elevated lysozyme production had increased gut permeability at steady-state and during infection, whereas *Lyz1*-deficient mice exhibit significantly elevated barrier functions. Although the growth and colonization of *Salmonella* are resistant to lysozyme *in vitro* and *in vivo*, proteomics and biochemical analysis revealed that engagement of lysozyme with *Salmonella* promoted the release of several known and poorly characterized virulence factors. We show that these factors impair the epithelial barrier *via* different mechanisms involving the activation of the Type Three Secretion System (T3SS) that builds translocons on the host cell membrane (18, 19, 20) as well as promoting epithelial cell TNFα production. Blocking lysozyme’s enzymatic action *via* inhibitor or denaturing temperature abolished its stimulation on the release of *Salmonella* virulence factor, attenuating the barrier impairment. Single-cell (sc)RNA-Seq analysis demonstrated that *Salmonella* infection robustly suppressed lysozyme RNA abundances in Paneth cells. We propose that the engagement of host intestinal lysozyme with an enteric pathogen such as *Salmonella* promotes the release of barrier-impairing factors; therefore, elevated or abnormal lysozyme production is pro-inflammatory during pathogenic bacterial infection.

## Results

### *S. Typhimurium* infection reduces lysozyme abundance in Paneth cells

**Figure 1:**
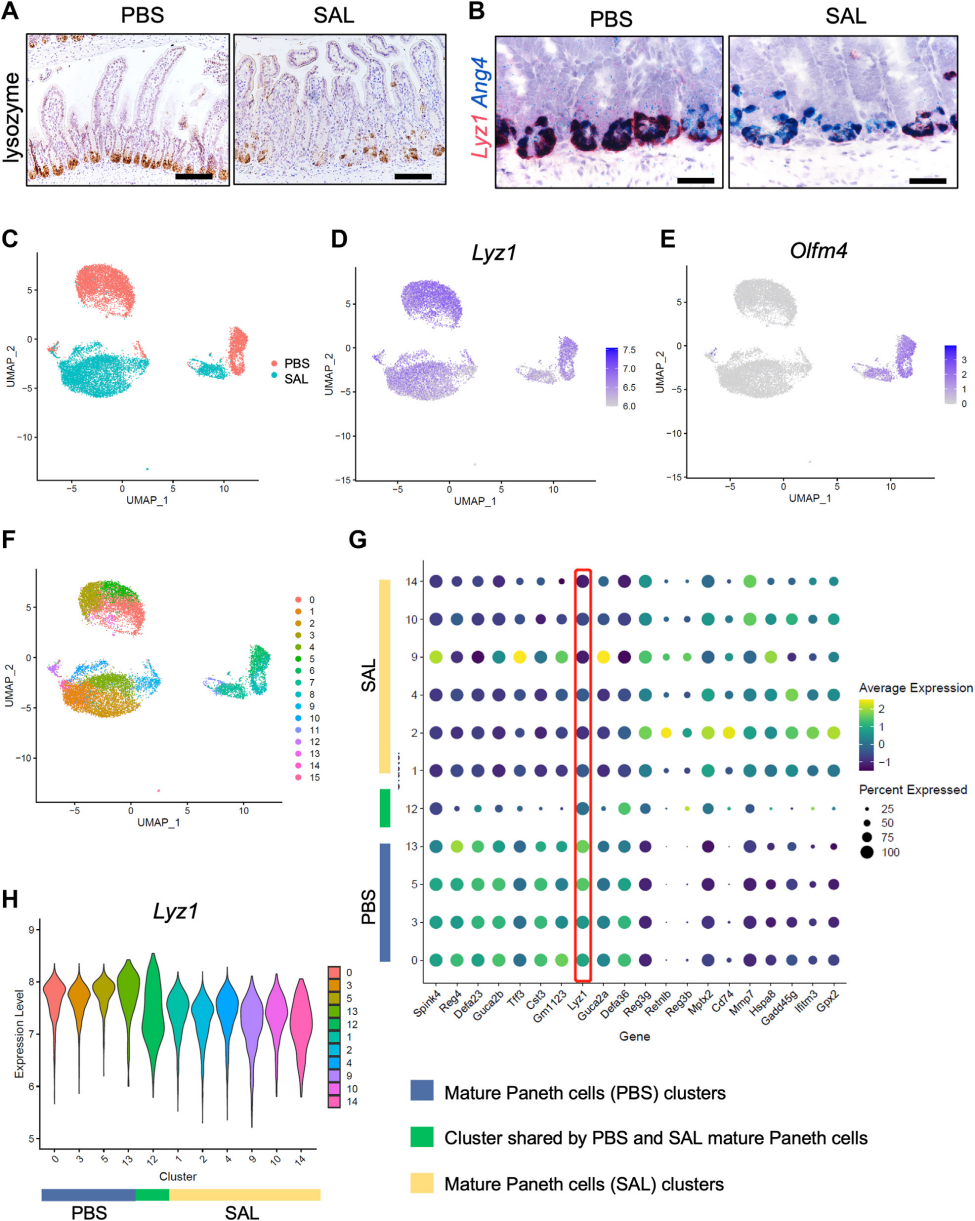
***S. Typhimurium* infection reduces lysozyme in Paneth cells.***A*, immunohistochemistry for lysozyme in uninfected and *Salmonella*-infected mouse ileum. *B*, dual RNA base scope for Lyz1 (*red*) and Ang4 (*blue*) in uninfected and *Salmonella*-infected mouse ileum. *C*, Seurat UMAP analysis of the single-cell transcriptome of 16,530 Paneth cells flow-sorted from uninfected (*red*) and *Salmonella*-infected (*blue*) Lyz1^3^′^UTR-IRES-CreER^; Rosa26R-dTomato^LSL^ mouse ileum. *D* and *E*, Seurat UMAP analysis for *Lyz1* and *Olfm4*. *F*, UMAP shows the clusters. Clusters 1, 2, 4, 9, 10, and 14 are mature Paneth cells in uninfected mice. Clusters 0, 3, 5, and 13 are mature Paneth cells in infected mice. Cluster 12 is mature Paneth cells shared by the two groups. Cluster 6, 7, 8, 11, clusters of Paneth cells progenitors. *G*, dot plots for Lyz1 (boxed in *red*) and other antimicrobial genes across the mature Paneth cell clusters. *H*, violin plot for Lyz1 across the mature Paneth cell clusters. The length of all scale bars in (*A* and *B*) are 100 μm.

Lysozyme is considered an antimicrobial peptide against bacterial infection (21). We first evaluated lysozyme expression in mice at homeostasis and after 10^8^ CFU *S. Typhimurium* (SL1344) infection *via* oral gavage. We found that infection reduced the lysozyme expression in Paneth cells at protein (Fig. 1*A*) and RNA levels (Fig. 1*B*). The reduction was not due to loss of Paneth cells as dual RNA base-scope analysis revealed a reduction of *Lyz1* (red) with a concomitant increase of *Ang4* (blue), a member of the ribonuclease A superfamily having an antimicrobial activity (22), in the infected mouse crypts compared to control mice inoculated by PBS (Fig. 1*B*).

To test if infection changes Lyz1 transcript abundance in a subpopulation of Paneth cells, we compared the scRNA profiles of ileal Paneth cells (SAL *versus* PBS) flow-sorted 4-days post-infection (4 dpi) (Fig. 1*C*) (23). UMAP for Lyz1 demonstrates a reduced transcript abundance in Paneth cells of infected mice (compare SAL to PBS, Fig. 1*D*). UMAP for Olfm4 distinguishes progenitor from mature Paneth cells (Fig. 1*E*), and there was a relatively more pronounced Lyz1 reduction in the mature Paneth cells of infected mice (Fig. 1*D*). Within the 11 mature Paneth cell clusters (Olfm4-negative) (Fig. 1, *E* and *F*), differential gene analysis shows that Lyz1 is significantly reduced in SAL specific clusters 1, 2, 4, 9, 10, and 14, when compared to PBS clusters 0, 3, 5, and 13 (red box, Fig. 1*G*). Cluster 12 is shared by PBS and SAL Paneth cells, where cells with both low and high Lyz1 abundances can be observed in the violin plots (Fig. 1*H*). In contrast to Lyz1, mature SAL clusters showed elevated abundances of *Reg3g, Mptx2*, and *Mmp7* (Fig. 1*G*), genes known to enrich in Paneth cells, especially during infection (23). Reg3g is a Paneth cell enriched antimicrobial peptide (24), Mptx2 is a Paneth cell mucosal pentraxin required for activation of lectin complement pathway (23, 25). Mmp7 is matrix metallopeptidase secreted by Paneth cells to process and activate pro-defensins (26). These data demonstrated an overall reduction of lysozyme in Paneth cells in response to infection, a change different from other Paneth cell-enriched antimicrobial genes.

### Intestinal epithelial lysozyme overexpression exacerbates *Salmonella*-induced inflammation

**Figure 2:**
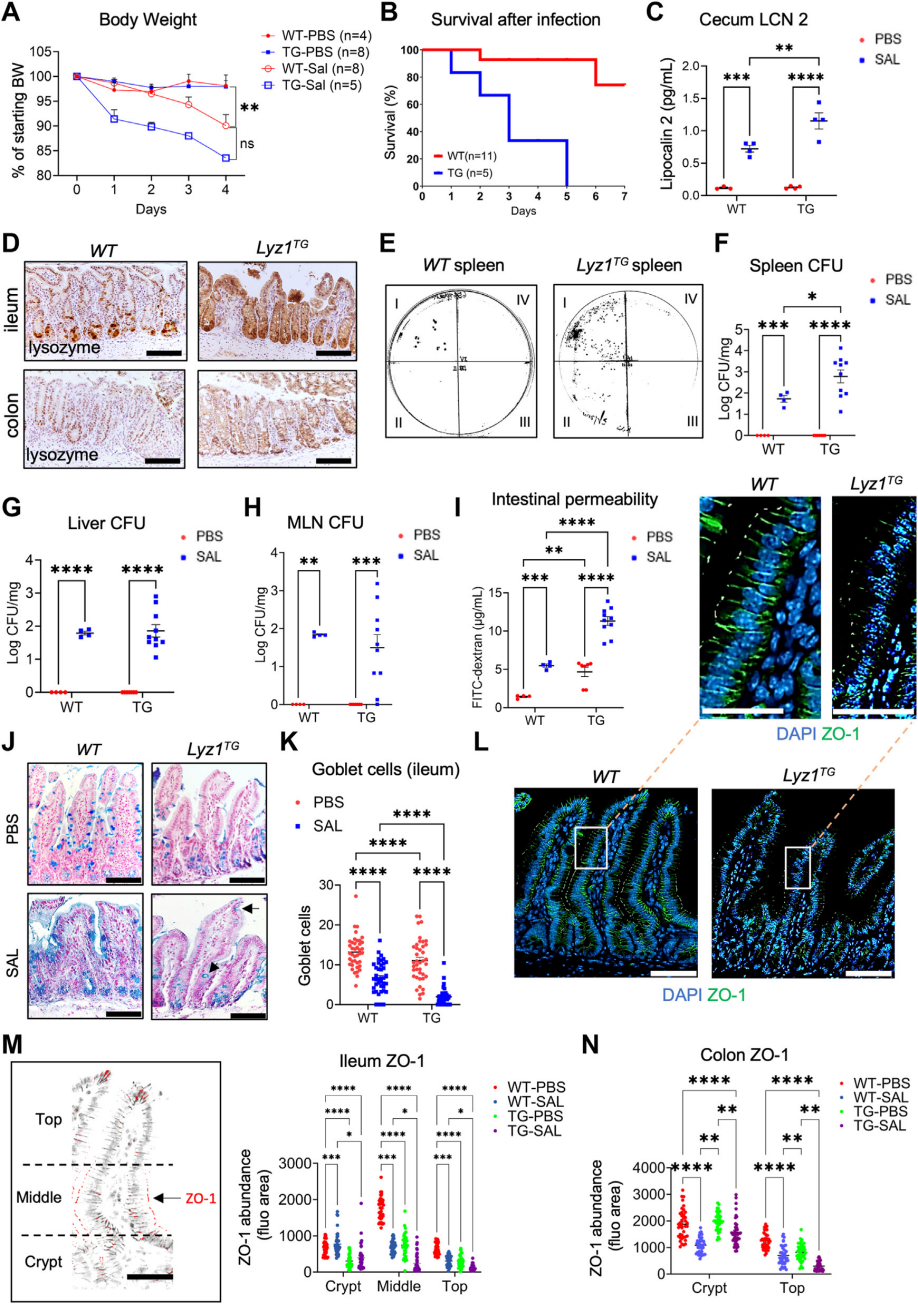
***Salmonella* infection caused more pronounced inflammatory disease in *Villin-Lyz1***^***TG***^**mice.***A*, mouse body weight change during 4 days post *Salmonella* infection. PBS or 10^8^ CFU of *Salmonella* were orally inoculated into WT or *Villin-Lyz1*^*TG*^ mice. Animal numbers for individual groups are specified in the graph. *B*, survival of WT or *Villin-Lyz1*^*TG*^ mice during 4 days post *Salmonella* infection. All samples analyzed below were collected from the survival mice on day 4. *C*, cecum lipocalin-2 (LCN-2) abundances were measured by ELISA from uninfected and infected WT and *Villin-Lyz1*^*TG*^ mice at sacrifice. *D*, immunohistochemistry for lysozyme in the infected distal ileum and colon in WT and *Villin-Lyz1*^*TG*^ mice. *E*, *Salmonella* CFU analysis in spleens from infected WT and *Villin-Lyz1*^*TG*^ mice at sacrifice. Every milligram of tissue was homogenized in 10 μl PBS. Region I represents a non-diluted homogenized tissue solution, while regions II to IV represent a 10-fold serial dilution of the previous tissue diluents. Each region had a triplicated 5μl-drop of the indicated dilutant. *F–H*, *Salmonella* CFUs in spleen, liver, and MLN of WT and *Villin-Lyz1*^*TG*^ mice at sacrifice. Each data point represents a biological replicate. Log_10_ of the exact CFUs was presented. *I*, *in vivo* permeability assay. Serum FITC concentrations (μg/ml) for individual biological replicates are plotted for WT and *Villin-Lyz1*^*TG*^ mice at sacrifice. *J* and *K*, representative alcian blue staining and quantification of goblet cells in the distal ileum of infected WT and *Villin-Lyz1*^*TG*^ mice. Each data point represents a crypt-villus unit scored from 6 to 10 different regions per animal. Five animals per genotype were analyzed. *L*, representative immunofluorescent staining for ZO-1 in ileum of WT and *Villin-Lyz1*^*TG*^ mice. *M*, quantitative analysis was based on different crypt-villus regions: crypt, middle of the villi, and villus tip, and obtained from uninfected and infected WT and *Villin-Lyz1*^*TG*^ mice. *N*, quantitative of ZO-1 in different colonic regions of uninfected and infected WT and *Villin-Lyz1*^*TG*^ mice. ∗*p* < 0.05, ∗∗*p* < 0.01, ∗∗∗*p* < 0.001, ∗∗∗∗*p* < 0.0001. The length of scale bars in (*D*, *J*, *L*, and *M*) are 100 μm. The length of scale bars in the L zoom magnification panels is 50 μm.

Within the mouse intestinal epithelium, *Lyz1* expression is restricted to Paneth cells (27, 28, 29). A transgenic line *Villin-Lyz1*^*TG*^ was previously developed by us to ectopically express lysozyme throughout the intestinal epithelial cells (6). To determine the impact of lysozyme overproduction on *Salmonella* infection, we inoculated *S. Typhimurium* (SL1344) to WT and *Villin-Lyz1*^*TG*^ mice (all on C57BL/6 background). Upon infection, *Villin-Lyz1*^*TG*^ mice demonstrated a significantly increased morbidity reflected by severe body weight loss (Fig. 2*A*), increased mortality (Fig. 2*B*), and elevated cecum lipocalin 2 (LCN2) (*p* = 0.0013, Fig. 2*C*), an indicator of inflammation (30). WT and *Villin-Lyz1*^*TG*^ mice that were inoculated with sterile PBS did not show a difference in body weight change (Fig. 2*A*) or in cecum LCN2 abundance (Fig. 2*C*).

Immunohistochemistry analysis demonstrated villus blunting in the ileum of infected *Villin-Lyz1*^*TG*^ mice (Fig. 2*D*). *Salmonella* colony formation unit (CFU) analysis detected a significantly increased *Salmonella* dissemination to the spleen of infected *Villin-Lyz1*^*TG*^ mice when compared to WT mice (*p* = 0.01, Fig. 2, *E* and *F*). Liver and intestinal mesenteric lymph node (MLN) of WT and *Villin-Lyz1*^*TG*^ mice showed equivalent *Salmonella* CFUs in WT and *Villin-Lyz1*^*TG*^ mice (Fig. 2, *G* and *H*), suggesting a preferential dissemination into the spleen of *Villin-Lyz1*^*TG*^ mice.

To determine if *Salmonella* induced different degrees of intestinal barrier disruption in WT and *Villin-Lyz1*^*TG*^ mice, we performed an *in vivo* gut permeability assay by gavaging FITC-dextran (60 mg/gram of BW) 4 h before euthanizing the mice for serum fluorescent analysis. Infected *Villin-Lyz1*^*TG*^ mice demonstrated a significantly higher intestinal permeability than WT mice (Fig. 2*I*). Interestingly, uninfected *Villin-Lyz1*^*TG*^ mice also exhibited a significantly elevated permeability than WT mice (*p* < 0.01, Fig. 2*I*), suggesting that the gut permeability in *Villin-Lyz1*^*TG*^ mice was already elevated before *Salmonella* infection whereas the infection further exacerbated the barrier impairment.

Abnormal permeability could be due to goblet cell reduction and epithelial mucin deficiency. Therefore, we examined the goblet cell population in *WT* and *Villin-Lyz1*^*TG*^ mice before and after infection. We found that in steady-state, *Villin-Lyz1*^*TG*^ mice have 25% fewer goblet cells per villus than WT mice (Fig. 2, *J* and *K*). *Salmonella* infection further reduced goblet cells in *Villin-Lyz1*^*TG*^ mice (Fig. 2, *J* and *K*). We noted a particularly diminished goblet cell population in the upper villus regions of infected *Villin-Lyz1*^*TG*^ mice (arrowhead, Fig. 2*J*).

We also examined tight junction (TJ) markers as their integrity is related to barrier function. Importantly, under steady-state conditions, ZO-1 is reduced in the ileum (Fig. 2, *L–M*), but not in the colon (Fig. 2*N*), of *Villin-Lyz1*^*TG*^ mice when compared to WT animals. After infection, there is a further reduction in ZO-1 in the ileal crypt, mid-villus, and upper villus epithelial regions (Fig. 2, *L–M*). Colonic epithelial ZO-1 was reduced in infected *WT* and *Villin-Lyz1*^*TG*^ mice, but the difference between the genotypes is insignificant (Fig. 2*N*). These results suggest a basal reduction in epithelial TJ proteins in *Villin-Lyz1*^*TG*^ mice, and this reduction was exacerbated by infection.

### *Lyz1* ablation protects against infection-induced gut inflammation

**Figure 3:**
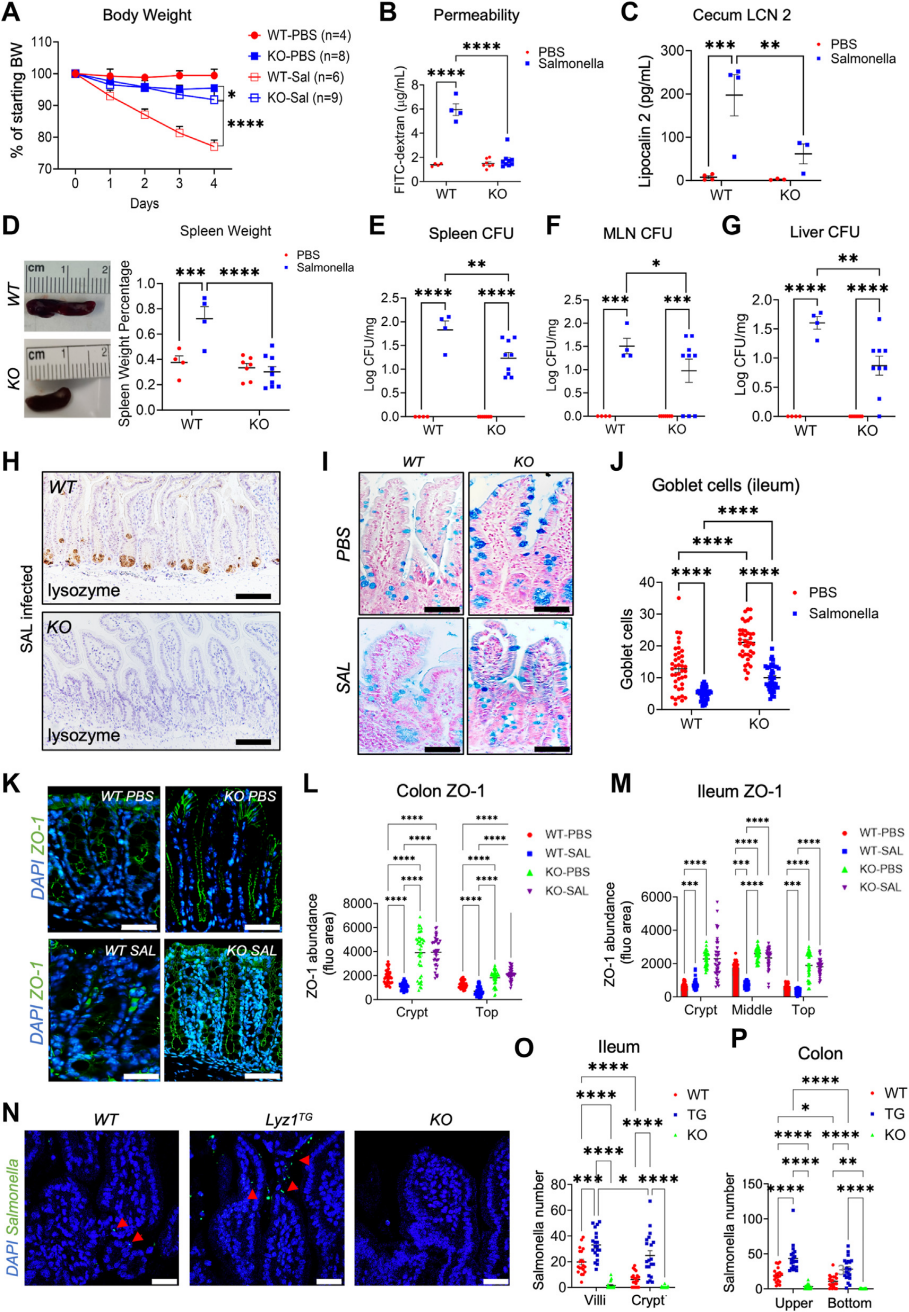
**Lyz1-deficient mice show attenuated inflammatory response to *Salmonella* infection.***A*, mouse body weight changes during the course of 4 days of *Salmonella* infection. PBS or 10^8^ CFU of *Salmonella* were orally inoculated to WT or Lyz1^−/−^ mice. *B*, intestinal permeability was measured as serum FITC-dextran concentration in uninfected and infected WT and Lyz1^−/−^ mice. All samples analyzed below were collected from the survival mice on day 4. *C*, cecum LCN-2 was measured in uninfected and infected WT and Lyz1^−/−^ mice at sacrifice. *D*, gross morphology of spleens and the percentage of spleen weights over body weights in uninfected and infected WT and Lyz1^−/−^ mice. *E–G*, *Salmonella* CFUs in the spleen, MLN, and liver of uninfected and infected WT and Lyz1^−/−^ mice. Log_10_ of the exact CFUs was presented. *H*, immunohistochemistry for lysozyme in infected WT and Lyz1^−/−^ mouse ileum. *I* and *J*, representative alcian blue staining and goblet cell quantification in distal ileum in WT and Lyz1^−/−^ mice. *K*, immunofluorescent staining for ZO-1 in distal colons of uninfected and infected WT and Lyz1^−/−^ mice. *L* and *M*, quantification of ZO-1 in colon and ileum in uninfected and infected WT and Lyz1^−/−^ mice. *N*, *Salmonella* staining in WT, *Villin-Lyz1*^*TG*^, and Lyz1^−/−^ mice. ∗*p* < 0.05, ∗∗*p* < 0.01, ∗∗∗*p* < 0.001, ∗∗∗∗*p* < 0.0001. The length of all scale bars in (*H*, *I*, *K*, and *N*) are 100 μm.

We then investigated how *Lyz1*^*−/−*^ mice, which lack the intestinal lysozyme (6), would respond to *Salmonella* infection. We performed the same infection procedures in WT and *Lyz1*^*−/−*^ littermate mice and monitored mouse body weight daily. Surprisingly, *Lyz1*^*−/−*^ mice do not exhibit a noticeable body weight loss in response to infection, as shown by *WT* mice, which had a 20% body weight reduction by day 4 (Fig. 3*A*). No mice died in these experiments. The protection against body weight loss seen in infected *Lyz1*^*−/−*^ mice was significant when compared to WT mice. FITC-dextran gut permeability analysis before sacrifice showed near total protection in infected *Lyz1*^*−/−*^ mice (Fig. 3*B*). Cecum LCN2 abundances in infected *Lyz1*^*−/−*^ mice were approximately 30% of WT mice (*p* = 0.009, Fig. 3*C*), while uninfected WT and *Lyz1*^*−/−*^ mice showed similar cecum LCN2 abundances (Fig. 3*C*).

To assess the progression of the systemic infection, spleen weight, and *Salmonella* CFU in the spleen were examined in infected *WT* and *Lyz1*^*−/−*^ mice at 4 d.p.i. The spleen-to-body weight ratio was 40% greater in *WT* mice than *Lyz1*^*−/−*^ mice (Fig. 3*D*). The reduced spleen weights reflect decreased systemic inflammation, consistent with a reduced *Salmonella* CFU per milligram of spleen tissues in *Lyz1*^*−/−*^ mice (Fig. 3*E*). Similarly, reduced CFUs were observed in *Lyz1*^*−/−*^ MLNs and livers (Fig. 3, *F* and *G*), but to a less extent.

Histopathology analysis suggested a much normal-appearing *Lyz1*^*−/−*^ intestinal epithelial morphology compared to *WT* mice (Fig. 3*H*), and a substantially increased number of goblet cells in *Lyz1*^*−/−*^ mice before and after infection (Fig. 3, *I* and *J*). Colonic epithelial cell ZO-1 abundances in *Lyz1*^*−/−*^ mice are also higher than in WT mice, before and after infection (Fig. 3, *K–L*). *Salmonella* infection reduced colonic ZO-1 in WT mice, but this reduction was not observed in *Lyz1*^*−/−*^ mice (Fig. 3*L*). ZO-1 abundances were overall higher in *Lyz1*^*−/−*^ ileal epithelial cells compared to WT mice (Fig. 3*M*).

As *Villin-Lyz1*^*TG*^ and *Lyz1*^*−/−*^ mice displayed contrasting phenotypes in response to *Salmonella* infection, we scored the visible number of invasive *Salmonella* in these mice (arrowheads in Fig. 3*N*). Compared to infected WT mice, *Salmonella* counts were elevated in *Villin-Lyz1*^*TG*^ mice but drastically reduced in *Lyz1*^*−/−*^ mice in both the ileum and colon (Fig. 3, *O* and *P*).

### Gut microbiota accounts in part for the increased permeability in *Villin-Lyz1*^*TG*^ mice at the steady state

**Figure 4:**
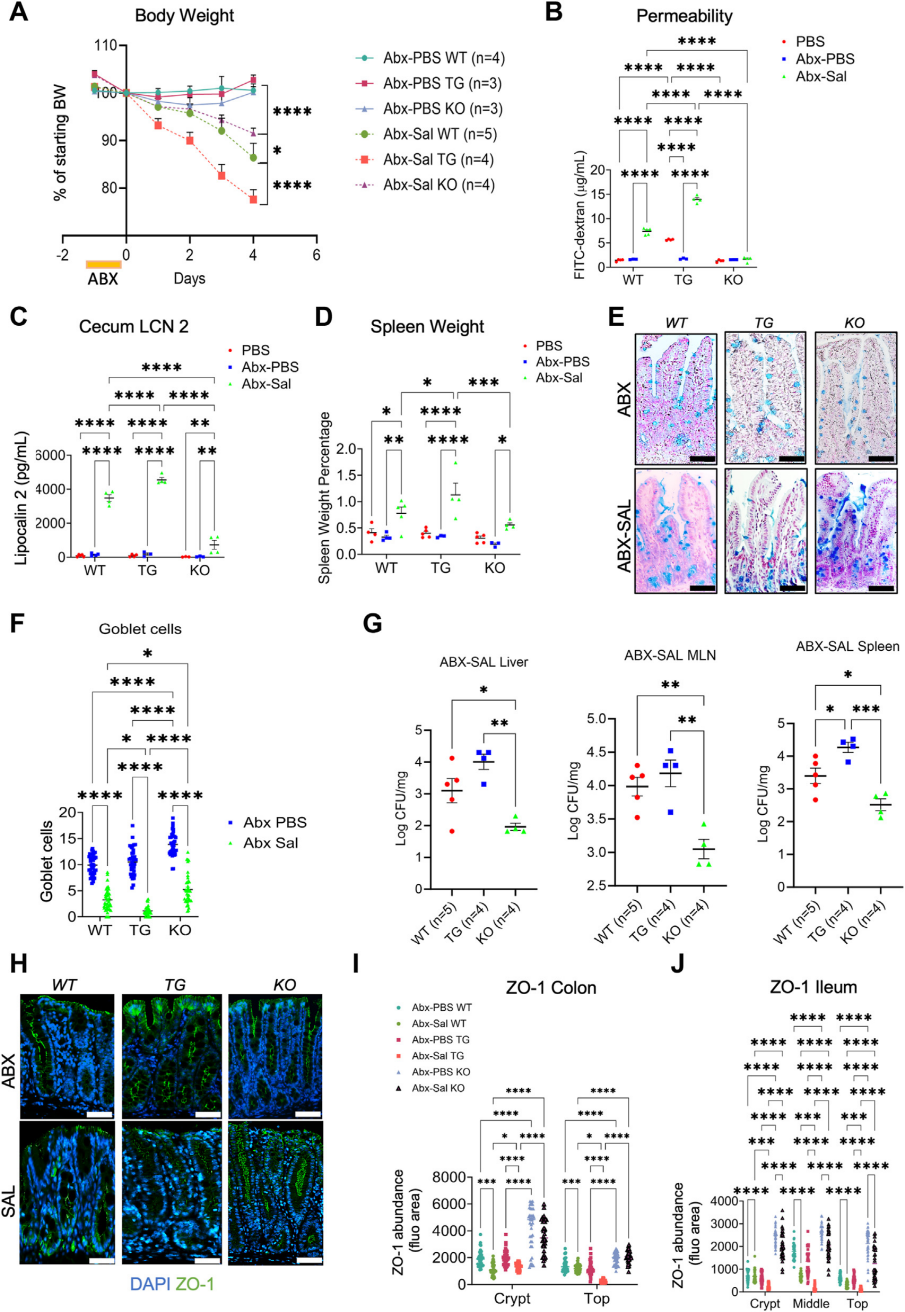
**Antibiotic treatment partially modifies *Villin-Lyz1***^***TG***^**and *Lyz1***^***−/−***^**mouse susceptibility to *Salmonella* infection.***A*, mouse body weight changes during 4 days of *Salmonella* infection. Mice were orally gavaged with antibiotics (Abx). After 1 day, mice were inoculated with *Salmonella* or PBS. All samples analyzed below were collected from the survival mice on day 4. *B*, intestinal permeability was measured based on serum FITC concentration at sacrifice. *C*, Cecum LCN-2 abundances in WT, *Villin-Lyz1*^*TG*^, and *Lyz1*^*−/−*^ mice treated with PBS, Abx, or Abx plus *Salmonella*. *D*, the percentage of spleen over total body weights. *E* and *F*, alcian blue staining and quantification of goblet cells in the distal ileum of WT, *Villin-Lyz1*^*TG*^, and *Lyz1*^*−/−*^ mice treated with Abx or Abx plus *Salmonella*. *G*, *Salmonella* CFUs in the liver, MLN, and spleen of infected WT, *Villin-Lyz1*^*TG*^, and *Lyz1*^*−/−*^ mice pretreated with Abx. Log_10_ of the exact CFUs was presented. *H*, representative ZO-1 staining of colons of WT, *Villin-Lyz1*^*TG*^, and *Lyz1*^*−/−*^ mice treated with Abx or with Abx plus *Salmonella*. *I* and *J*, quantifications of ZO-1 in the colon or ileum of the above mice. ∗*p* < 0.05, ∗∗*p* < 0.01, ∗∗∗*p* < 0.001, ∗∗∗∗*p* < 0.0001. The length of all scale bars in (*E* and *H*) are 100 μm.

Gut microbiota landscapes are changed in *Villin-Lyz1*^*TG*^ and *Lyz1*^*−/−*^ mice (6). To assess whether the changed microbiota might have contributed to the observed infection susceptibility in *Villin-Lyz1*^*TG*^ and *Lyz1*^*−/−*^ mice, we administrated mice with antibiotics (Abx) in drinking water to disrupt the microbiota, then challenged these mice with oral *Salmonella* inoculation. Overall, Abx-pretreated *Villin-Lyz1*^*TG*^ mice remained highly susceptible to infection, whereas Abx-pretreated *Lyz1*^*−/−*^ mice remained protected from the infection, based on body weight changes following *Salmonella* challenge (*p* = 0.042, WT *versus* KO, Fig. 4*A*). However, Abx-pretreatment appeared to partially reduce the protection observed in *Lyz1*^*−/−*^ mice, suggesting that the preexisting microbiota in these mice may have partially contributed to the protective role (Fig. 4*A*). Interestingly, gut permeability assay showed that the increased permeability in *Villin-Lyz1*^*TG*^ mice was diminished by Abx pretreatment (compare red and blue for TG, Fig. 4*B*), suggesting that the preexisting gut microbiota in *Villin-Lyz1*^*TG*^ mice to some extent was responsible for the elevated permeability at steady-state in these mice. However, upon challenge by *Salmonella*, the permeability was drastically elevated in these mice (compare blue to green for TG, Fig. 4*B*). In contrast, *Lyz1*^*−/−*^ mice exhibited minimal changes in gut permeability in response to Abx treatment or to the infection (Fig. 4*B*). These responses were largely reflected in cecum LNC2 abundances (Fig. 4*C*), spleen weights (Fig. 4*D*), goblet cell counts (Fig. 4, *E* and *F*), and *Salmonella* CFU in liver, MLN, and spleen (Fig. 4*G*). Immunofluorescent analysis for ZO-1 in the colon and ileum showed that Abx-treated *WT* and *Villin-Lyz1*^*TG*^ mice have similar ZO-1 abundances at steady states (Fig. 4, *H–J*). However, there was a significant infection-induced ZO-1 loss in Abx-pretreated WT and *Villin-Lyz1*^*TG*^ mice; and this reduction was not seen in the *Lyz1*^*−/−*^ mice (Fig. 4, *H–J*).

Thus, Abx treatment of *Villin-Lyz1*^*TG*^ mice improved gut permeability at the steady state, suggesting that the increased lysozyme engagement with gut microbiota in these mice may elicit an enhanced epithelial permeability. However, in terms of *Lyz1*^*−/−*^ mice, disrupting microbiota by antibiotics did not drastically alter the protection observed in these mice against disease phenotypes during infection. These data overall point to a pro-inflammatory role of intestinal lysozyme in *Salmonella* infection.

### Direct processing of *Salmonella* by lysozyme produces barrier-disrupting molecules

**Figure 5:**
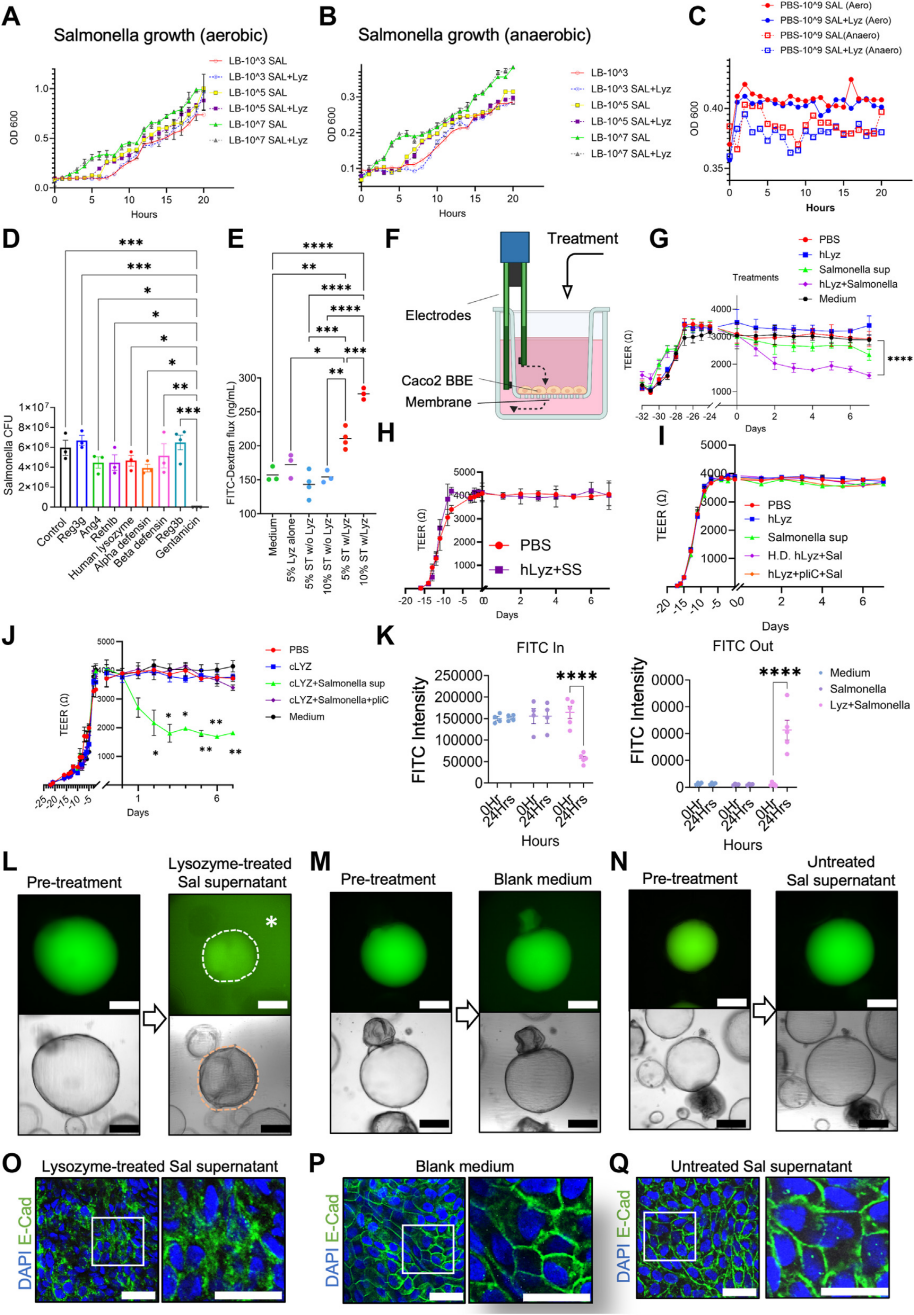
**Lysozyme-processed *Salmonella* produces barrier-disrupting molecules.***A*, *Salmonella* growth rate curve, reflected as OD600 readings, under aerobic conditions in the presence or absence of 20 μg/ml lysozyme. Cells were seeded at different numbers at initial time points. *B*, *Salmonella* growth rate curve, reflected as OD600 readings, under anaerobic conditions in the presence or absence of 20 μg/ml lysozyme. Cells were seeded at different numbers at initial time points. *C*, *Salmonella* was resuspended in PBS with or without lysozyme under aerobic or anaerobic conditions. OD600 readings were measured on a time course. *D*, Caco2 BBE cells were grown to differentiated monolayers and maintained for 2 weeks. Individual antimicrobial proteins were added for 15 min at concentrations described in Methods. *Salmonella* SL1344 was added to cells at a MOI of 100 and incubated with cells for 30 min to allow invasion at 37 °C, 5% CO2. Cells were then washed with 150 μg/ml gentamicin (Corning, #30-005-CR) for 50 min to remove extracellular bacteria. Cells were lysed and the intracellular *Salmonella* CFUs were determined from the lysates on XLD agar plates. *E*, Caco2 monolayers were seeded on trans-wells, and apical medium containing 10 mg/ml 4 kDa FITC-dextran (Sigma, FD4) was supplemented with 5 or 10% of filtered-sterilized LB broth from (i) 16 h *Salmonella* culture without lysozyme, (ii) LB broth with 4000 U/ml of human lysozyme without bacteria, (iii) conditioned medium from *Salmonella* culture treated with 4000 U/ml of human lysozyme for 16 h. *F*, Schematic diagram for TEER measurement. Treatments were applied to the apical side of the Caco2 BBE monolayer. *G*, TEER readings of monolayer Caco2 BBE in 96-well-plate before and after treatment with cell culture medium, PBS (vehicle), 0.2 μg/ml human lysozyme alone in PBS, 10% untreated *Salmonella* supernatant or supernatants from *Salmonella* treated by 0.2 μg/ml human lysozyme in PBS. *H*, TEER readings of monolayer Caco2 BBE in 96-well-plate before and after treatment with PBS (vehicle), or *Salmonella* supernatants (SS) preincubated with 0.2 μg/ml human lysozyme. *I*, TEER readings of monolayer Caco2 BBE in 96-well-plate before and after treatment with PBS, 0.2 μg/ml lysozyme alone in PBS, *Salmonella* supernatants, supernatant of lysozyme-treated *Salmonella* in the presence of 1 μg/ml pliC, supernatant from *Salmonella* treated by heat-denatured (H.D.) 0.2 μg/ml human lysozyme. *J*, TEER readings of monolayer Caco2 BBE in 96-well-plate before and after treatment with cell culture medium, PBS (vehicle), 0.2 μg/ml chicken lysozyme alone in PBS, 10% untreated *Salmonella* supernatant or supernatants from *Salmonella* treated by 0.2 μg/ml chicken lysozyme in PBS, supernatant of lysozyme-treated *Salmonella* in the presence of 1 μg/ml pliC. *K*, quantification of FITC fluorescent intensities inside and outside of enteroids with luminally injected FITC-dextran, and subsequently treated 24 h with blank *Salmonella* culture medium, *Salmonella* spent medium, and lysozyme-treated *Salmonella* medium supernatants. *L–N*, mature enteroids were luminally injected with FITC-dextran, then 3 groups of enteroids were treated with blank *Salmonella* culture medium, *Salmonella* spent medium, and lysozyme-treated *Salmonella* medium supernatants for 24 h. Images were taken pre and after treatments. FITC intensities inside and outside (indicated by an *asterisk* in *K*) were quantified and shown in (*J*). *O–Q*, Representative immunofluorescent analysis for E-Cadherin and DAPI in the above treated enteroids. ∗*p* < 0.05, ∗∗*p* < 0.01, ∗∗∗*p* < 0.001, ∗∗∗∗*p* < 0.0001. The length of scale bars in (*L–N*) is 100 μm, and in (*O–Q*) is 50 μm.

We tested *in vitro* if lysozyme affects *Salmonella’s* growth or invasiveness toward enterocytes. We plotted the growth curve of *Salmonella* under aerobic (Fig. 5*A*) and anaerobic (Fig. 5*B*) conditions over 20 h, in the presence or absence of human lysozyme. We used a lysozyme concentration of 20 μg/ml which is approximately 50 to 200 times of lysozyme concentrations in the secretions (31). Regardless of the initial seeding of *Salmonella* numbers, *Salmonella* grew at a similar rate in the presence or absence of lysozyme (Fig. 5, *A* and *B*). When we incubated 10^9^ CFU *Salmonella* with the presence or absence of lysozyme in PBS, the *Salmonella* did not change throughout 20 h (Fig. 5*C*), suggesting that lysozyme did not kill *Salmonella* in PBS under aerobic or anaerobic conditions. These data suggest that human lysozyme does not affect *Salmonella* growth or death.

To test if lysozyme may alter *Salmonella*’s invasiveness towards human enterocytes, we infected Caco2 BBE monolayers with *Salmonella* at a MOI of 250 in the presence of human lysozyme preincubated with the cells. After 40 min of infection, we quantified the intracellular *Salmonella* by CFU assays. Lysozyme preincubation did not significantly reduce *Salmonella’s* intracellular invasiveness (Fig. 5*D*). Similar observations were observed with the preincubation of cells with Reg3b, Reg3g, Ang4, Retnlb, α-defensin, or β-defensin, which are antimicrobial peptides enriched in Paneth and goblet cells, and have been previously associated with bactericidal functions and gastrointestinal inflammation (32, 33, 34). However, gentamycin preincubation with the cells completely diminished *Salmonella* invasiveness (Fig. 5*D*), serving as a positive control. Thus, lysozyme does not alter *Salmonella*’s growth capacity or invasiveness *in vitro*.

To assess the above observation in a 2-D monolayer setting, we performed FITC-dextran permeability assays on Caco2 BBE monolayers using conditioned media from *Salmonella* culture at 5 or 10% concentration in the apical medium (Fig. 5*E*). Blank *Salmonella* culture medium, medium with 4000 U/ml of human lysozyme without bacteria, or conditioned medium from *Salmonella* culture with 4000 U/ml of human lysozyme. After 24 h of treatment, we found that compared to the control medium group, lysozyme alone or supernatants from untreated *Salmonella* supernatants did not alter the FITC-dextran permeabilities (Fig. 5*E*). However, lysozyme-pretreated *Salmonella* supernatants increased permeability in a concentration-dependent fashion (*p*_5%_ = 0.0029, *p*_10%_ < 0.0001, Fig. 5*E*).

We then directly measured the Trans-Epithelial Electrical Resistance (TEER) of the Caco2 BBE monolayer on a 96 trans-well plate (Fig. 5*F*). Two days following the addition of human lysozyme-treated *Salmonella* supernatants TEER was significantly reduced, whereas TEER was not altered in monolayers treated by lysozyme alone or by untreated *Salmonella* supernatants (Fig. 5*G*). To distinguish if lysozyme acted on *Salmonella* directly or on molecules in the *Salmonella* supernatant (SS) to invoke the barrier disruption, we treated *SS* with lysozyme, then applied the treated mixture to Caco2 BBE monolayer. No TEER reduction was detected (Fig. 5*H*). When we added 1 μg/ml periplasmic lysozyme inhibitor of c-type lysozyme (pliC), to the lysozyme and *Salmonella* mixture, no significant TEER reduction was observed (Fig. 5*I*). In addition, when we used heat-denatured (HD) lysozyme to treat *Salmonella*, the resulting supernatants also did not induce TEER change (Fig. 5*I*), collectively supporting that lysozyme directly engages with live *Salmonella* potentially *via* its catalytic function to impair the epithelial barrier. Furthermore, 0.2 μg/ml chicken lysozyme-treated *SS* also decreased the Caoc2 TEER (Fig. 5*J*), and pliC also inhibited the decrease (Fig. 5*J*).

To further test if lysozyme-treated *Salmonella* may release barrier-degrading factors, we collected supernatants from human lysozyme-treated and untreated *Salmonella* and tested their effects on epithelial permeability in human enteroids. FITC-dextran was microinjected into the enteroid lumen, followed by treating the enteroids with lysozyme-treated (Fig. 5*L*), blank *Salmonella* culture medium (Fig. 5*M*), or untreated *Salmonella* spent medium (Fig. 5*N*). After 24 h of treatment, FITC-dextran was observed to leak out from the enteroids treated with lysozyme-treated *SS* (asterisk, Fig. 5*L*). The FITC-dextran signals were measured inside of the lumen and outside of the enteroid (Fig. 5*K*). The functional integrity of these treated enteroids was analyzed by immunofluorescent staining for E-Cadherin (Fig. 5, *O–Q*), and cytosolic localization of E-Cadherin in Lyz+Sal treated enteroids reflected the increased permeabilities (Fig. 5, *K–M*).

### Proteomic identification of lysozyme-induced release of *Salmonella* virulence factors

**Figure 6:**
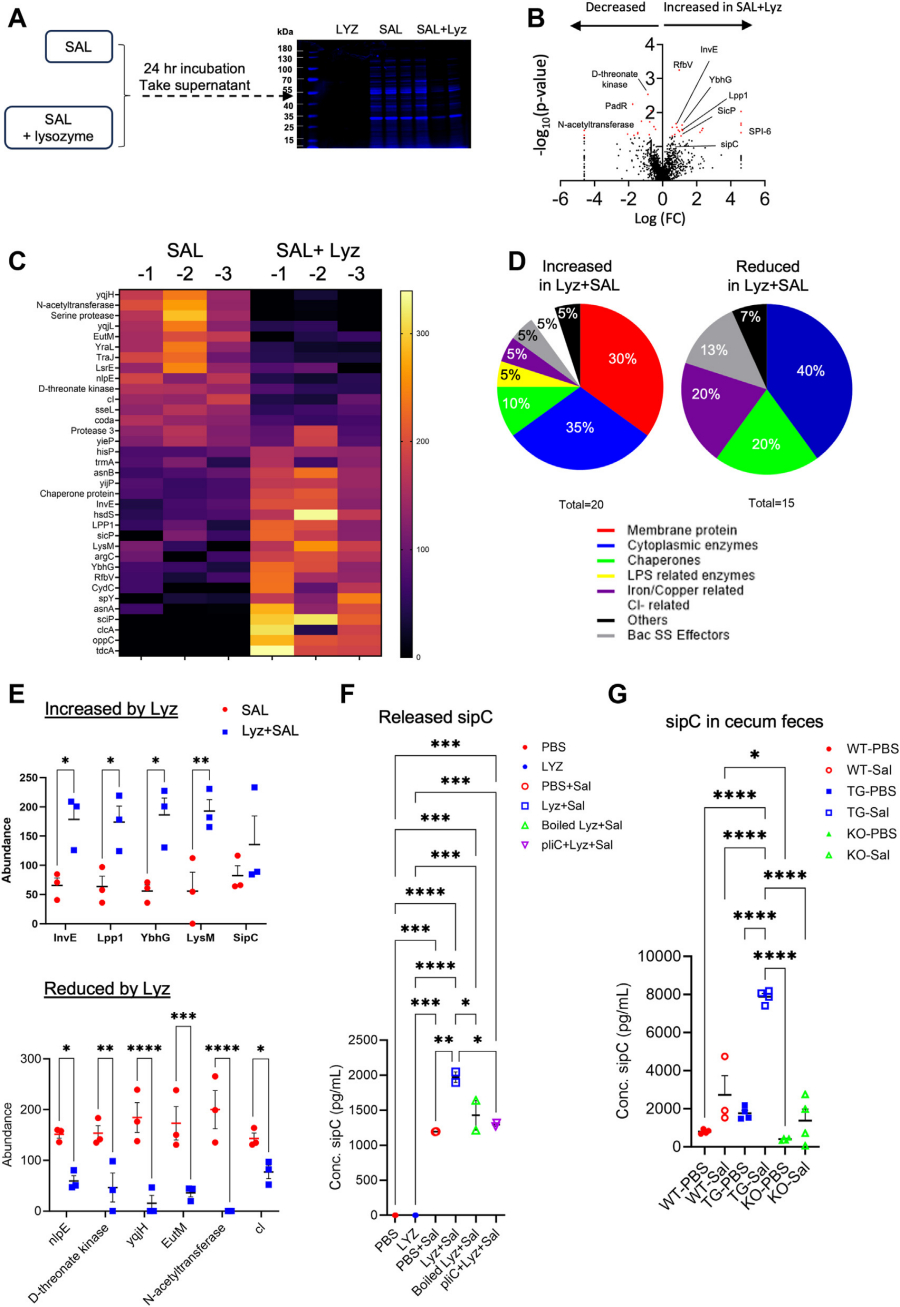
**Proteomic identification of lysozyme-stimulated release of *Salmonella* virulent factors.***A*, supernatants from untreated *Salmonella* and *Salmonella* treated with 0.2 μg/ml lysozyme, as well as 0.2 μg/ml lysozyme alone in PBS were resolved on SDS-PAGE. Protein bands were stained and cut for each sample and subjected to proteomic analysis by mass spectrometry. Three biological replicates were used for each condition. *B*, increased and decreased proteins were plotted on a volcano plot, with the red dots representing targets with more than 1.3-fold change and *p*-value < 0.05, between Sal *versus* Lyz+Sal groups. Log_10_ of the exact fold changes, and -Log_10_ of *p*-Value were presented. *C*, Heatmap of 35 significantly changed proteins. *D*, functional categorizations of increased and decreased protein targets in Lyz+Sal groups compared to Sal alone. *E*, the top increased and decreased proteins in the supernatant of lysozyme-treated *Salmonella*. The abundance of a peptide is measured by the total number of MS/MS peptide spectra matches (PSMs, please also see Table 1). Each point represents the protein abundance based on PSM in each sample. *F*, sipC ELISA analysis from PBS, lysozyme alone, *Salmonella* supernatants, supernatants from *Salmonella* treated with 0.2 ug/ml lysozyme, supernatants from *Salmonella* treated with heat-denatured lysozyme, and supernatants from *Salmonella* treated with lysozyme in presence of recombinant pliC. *G*, sipC ELISA analysis from cecum feces from uninfected and *Salmonella-infected WT*, *Villin-Lyz*^*TG*^, and *Lyz*^*−/−*^ mice. ∗*p* < 0.05, ∗∗*p* < 0.01, ∗∗∗*p* < 0.001, ∗∗∗∗*p* < 0.0001.

We next performed an untargeted proteomic profiling to investigate the identities of molecules that are present in lysozyme-treated *Salmonella* supernatants. *Salmonella* can survive in PBS for up to 30 weeks (35). We, therefore, prepared three conditions: (i) live *Salmonella* were spun down and resuspended in PBS containing 0.2 μg/ml human lysozyme, (ii) live *Salmonella* were spun down and resuspended in PBS without lysozyme, and (iii) 0.2 μg/ml human lysozyme in PBS. Samples in triplicates were incubated for 24 h at 37 °C, collected, resolved by SDS-PAGE, and subjected to in-gel digestion and proteomic analysis by mass spectrometry (Fig. 6*A*). The proteomic analysis identified a total of 1961 proteins, however, only 35 proteins were differentially present in lysozyme-processed *versus* un-processed *Salmonella* (*p*-value < 0.05, 20 increased and 15 decreased) (Fig. 6, *B* and *C* and Table 1). Compared to untreated *Salmonella*, 30% of the increased proteins in lysozyme-treated *Salmonella* supernatants are membrane proteins, 35% are enzymes, while 10% and 5% are chaperones and LPS related enzymes (Fig. 6*D*). The majority of reduced proteins are enzymes, chaperones, and iron or chlorine related proteins (Fig. 6*D*).

**Table 1:** 35 significantly changed proteins

Protein name	Protein ID	Coverage (%)	Spectrum counts	Unique peptides	Amin acids	Molar weights	SAL abundance	LYZ+SAL Abundance	Fold changes	*p*-Value	Function
Siderophore- interacting protein	yqjH	10.98	4	3	255	28.9	184.4333	0	0	0.019824	Ferric reductase in different iron assimilation pathways
N-acetyltransferase		21.71	3	3	175	19.1	200	0	0	0.033553	Transfer actyl group from intercellular into bacteria
Cytoplasmic protein		22.37	3	1	76	8.3	200	0	0	0.046521	Cytoplasmic proteins
Serine protease		11.72	6	3	273	29.1	173.9667	14.86667	0.1	0.042902	Hydrolaysis serine
PadR family transcriptional regulator	yqji	14.88	9	3	215	24.2	173.5667	26.43333	0.2	0.005656	Represses the expression of YqjH which is involved in iron homeostasis under excess nickel conditions.
Ethanolamine utilization microcompartment protein EutM	EutM	37.5	11	3	96	9.8	173.1333	26.86667	0.2	0.046791	A transcriptional repressor, is important for preventing expression when other preferred sources of energy are available
Ribosomal RNA small subunit methyltransferase I	yraL	24.74	15	6	287	31.5	173.3	26.7	0.2	0.042308	Active on the assembled 30S subunit
Conjugal transfer protein, ATP-binding	traJ	14.4	14	5	382	42.5	173.1	26.9	0.2	0.038769	Activates the PY promoter and the transcription of the operon
Putative epimerase LsrE	LsrE	19.29	11	4	254	27.6	159	41	0.3	0.01799	Putative epimerase, is the final protein in the A-2 quorum sensing pathway yet to be characterize
Copper homeostasis protein CutF (Lipoprotein nlpE)	nlpE	9.01	9	3	233	25.2	151.2333	48.73333	0.4	0.002936	Involved in both copper efflux and the delivery of copper to copper-dependent enzymes
D-threonate kinase		18.68	10	6	423	45	153.2667	46.7	0.5	0.044597	A kinase involved in the catabolic pathway of D-threonate
Bacteriophage repressor protein cI	cl	19.37	11	3	191	20.5	143.2333	56.73333	0.5	0.018677	Prevents lytic growth by directly repressing two promoters needed to express lytic functions, P_L_ and P_R_
Type III secretion system effector protein, deubiquitinase	sseL	16.4	18	5	317	35.5	140.9	59.1	0.5	0.008821	Translocated into host cells during intracellular infection
Cytosine deaminase	codA	34.98	46	11	426	47.6	137.5667	62.46667	0.6	0.025808	Allows the cell to utilize cytosine for pyrimidine nucleotide synthesis
Protease 3		15.7	56	13	962	107.4	133.3	66.7	0.7	0.031043	Endopeptidase (alkaline protease) of the serine protease family and cleaves proteins in the tissue section
FadR family transcriptional regulator	yieP	23.58	7	5	229	26	81.43333	118.5667	1.8	0.027262	Regulates the expression of genes encoding fatty acid biosynthetic and degrading enzymes
Histidine transport ATP-binding protein	hisP	14.34	9	3	258	28.8	80.9	119.1	1.8	0.048651	Part of the ABC transporter complex HisPMQJ involved in histidine transport
tRNA/tmRNA (uracil- C(5))-methyltransferase	trmA	20.49	19	6	366	41.9	78.23333	121.7333	2.1	0.043658	Dual-specificity methyltransferase that catalyzes the formation of 5- methyluridine at position 54 (m5U54) in all tRNAs, and that of position 341 in tmRNA
Asparagine synthase B	asnB	25.27	52	13	554	62.5	71.03333	128.9667	2.2	0.021078	Catalyzes the ATP- dependent conversion of aspartate into asparagine, using glutamine as a source of nitrogen
Hypothetical membrane protein	yijP	11.36	10	2	132	14.8	69.63333	130.3333	2.3	0.027148	Membrane protein
Cell invasion protein	invE	18.82	17	6	372	42.4	65.36667	134.6	2.5	0.032865	Triggering of intracellular events that lead to microbial internalization
Type I restriction enzyme	hsdS	8.1	10	3	469	51.9	63.86667	136.1667	2.6	0.000564	Recognizes the target DNA sequence
Major outer membrane lipoprotein Lpp	Lpp1	62.82	85	6	78	8.4	63.6	136.3667	2.7	0.000564	Major outer membrane
Chaperone protein SicP	slcP	6.9	3	1	116	12.9	58.86667	141.1667	3	0.03567	Binds SptP, which in its absence completely is degraded within tie bacterial cytoplasm
Peptidoglycan-binding protein LysM	lysM	52.35	17	6	149	16.1	55.73333	144.2667	3.1	0.048491	Outer membrane protein, activates a common signal transduction pathway response
N-acetyl-gamma-glutamyl-phosphate reductase	argC	7.19	4	2	334	35.9	55.4	144.6	3.1	0.031262	Catalyze NADPH- dependent reductive dephosphorylation of N-acetyl-gamma- glutamyl-phosphate to N-acetylglutamate- gamma-semialdehyde
UPF0194 membrane protein YbhG	YbhG	45.32	34	12	331	36.3	55.96667	144.0667	3.2	0.023133	Membrane protein
Abequosyltransferase RfbV	RfbV	16.22	17	6	333	38.6	56.36667	143.6333	3.3	0.033607	Catalyzes the transfer of CDP-abequose on D-mannosyl-L- rhamnosyl-D- galactose-1- diphospholipid to yield D-abequosyl-D-mannosyl-rhamnosyl-D-galactose-1-diphospholipid
Cysteine/glutathione ABC transporter ATP- binding protein/permease CydC	CydC	15.36	12	6	573	63	22.33333	177.6667	9	0.032605	It is involved in the export of glutathione from the cytoplasm to the periplasm and is required for the assembly of both cytochrome c and cytochrome bd
ATP-independent periplasmic protein-refolding chaperone	spy	16.15	5	3	161	18.2	20.66667	179.3333	10	0.03805	Folding landscape of the substrate in determining chaperone mechanisms
Aspartate--ammonia	asnA	17.88	11	5	330	36.8	22.76667	177.2667	10.6	0.033566	Catalyzes the ATP- dependent conversion of Asn
Hypothetical membrane protein (SPI- 6 associated)	sclP	1.61	2	1	434	47.3	0	200	100	0.029191	Membrane protein
H(+)/Cl(−) exchange transporter ClcA	clcA	2.75	1	1	473	50.4	0	200	100	0.009301	Proton-coupled chloriode transporter
Oligopeptide ABC transporter permease OppC	oppC	7.95	2	2	302	33.1	0	200	100	0.020463	Binds peptides of 3 amino acids to at least 16 amino acids in length independent of amino acid content
TDC operon transcriptional activator	tdcA	6.73	7	2	312	34.8	0	200	100	0.023254	Membrane protein

Spectrum counts (SC) and unique peptides (UP) were utilized to rank the confidence scores (Table 1). Increased membrane proteins with the highest confidence scores and were consistently represented in all replicates include InvE (*p* = 0.033), Lpp1 (*p* = 0.036), UPF 0194 (YbhG) (*p* = 0.034), and LysM (*p* = 0.048) (Fig. 6*F*). InvE is a cell invasion protein that recruits sipB and sipC to form a translocon complex (20), and shows 2.6-fold increase in lysozyme-processed *Salmonella* supernatants (Fig. 6*E*). Lpp1 is a major outer membrane protein reported to stimulate TNFα production (36), and shows 2.7-fold increase. YbhG is an uncharacterized membrane protein associated with resistant chloroamphenicol and virulence production (36, 37, 38), and shows 3.2-fold increase. LysM, a liposaccharide binding protein linked to virulence by recognizing the enzymatic target, GlcNAc-X-GlcNAc (39), shows 3.1-fold increase.

sipC can be recruited by InvE in the *Salmonella* T3SS and forms a translocon complex with sipB on the host cell membrane with the help of other effectors (20). As sipC shows borderline significance in our proteomic analysis (Fig. 6*E*), we performed ELISA analysis for sipC to directly measure its abundances in lysozyme-treated *versus* untreated *Salmonella* supernatants. We found that lysozyme treatment significantly stimulated *Salmonella* to release sipC to the supernatants (Fig. 6*F*). Such induction of sipC was diminished when *Salmonella* was treated with heat-denatured lysozyme or in the presence of lysozyme inhibitor, pliC (Fig. 6*F*). To extent this observation to *in vivo* analysis, we measured sipC in the cecum feces of WT, *Villin-Lyz1*^*TG*^ and *Lyz1*^*−/−*^ mice. Interestingly, under the steady-state condition (without infection), *Villin-Lyz1*^*TG*^ mice showed slightly elevated sipC in the cecum contents (Fig. 6*G*). After infection, there was an increased sipC in all groups, but the *Villin-Lyz1*^*TG*^ mice demonstrated the most robust elevation of sipC (*p* < 0.0001, Fig. 6*G*). These results suggest that lysozyme engagement with commensal bacteria may contribute to basal level of sipC production, and that lysozyme interaction with *Salmonella* during infection triggers the release of more sipC from the pathogen.

### InvE, Lpp1, and sipC impair the intestinal epithelial barrier

To investigate if any of the above lysozyme-stimulated *Salmonella* factors may impair barrier integrity, we functionally examined InvE, Lpp1, YbhG, and sipC in TEER assays. Caco2 BBE monolayers were apically treated with individual factors at a concentration gradient of 0.1, 0.5, 1.0, or 5.0 μg/ml, each with 4 replicates. TEER readings were collected every day before and after the treatment.

**Figure 7:**
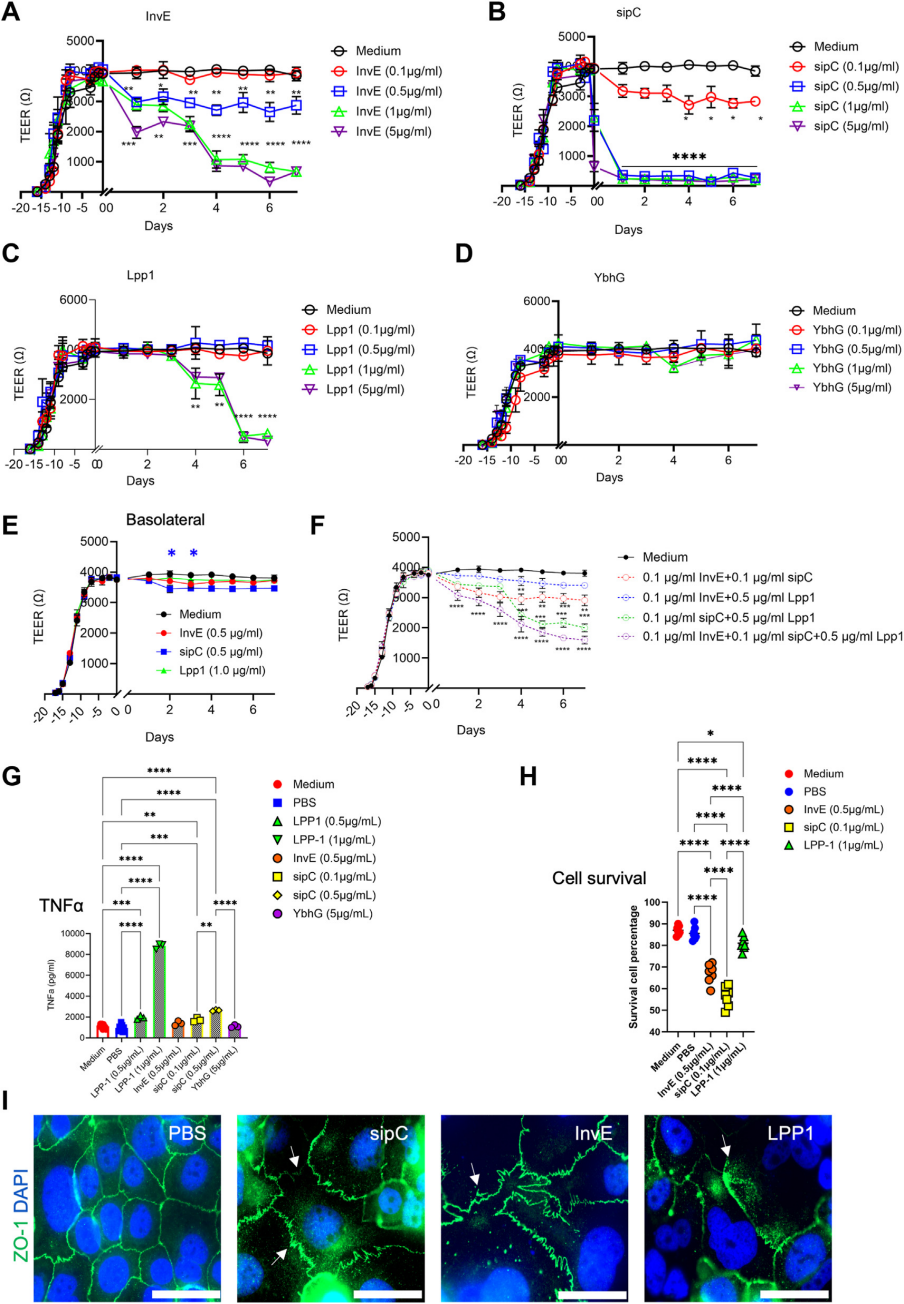
**InvE, sipC, and Lpp1 impair barrier function.***A*, TEER readings of Caco2 BBE monolayer in a 96-well plate before and after the treatment with increasing concentrations of InvE, applied apically. *B*, TEER readings of Caco2 BBE monolayer in a 96-well plate before and after the treatment with increasing concentrations of sipC, applied apically. *C*, TEER readings of Caco2 BBE monolayer in a 96-well plate before and after the treatment with increasing concentrations of Lpp1, applied apically. *D*, TEER readings of Caco2 BBE monolayer in 96-well plate before and after the treatment with increasing concentrations of YbhG, applied apically. Note, that the medium control for (*A–D*) was used as the baseline for all these individual treatments ran in the same experiments. *E*, TEER readings of Caco2 BBE monolayer in 96-well-plate before and after the treatment with 0.5 μg/ml InvE, or 0.5 μg/ml sipC, or 1.0 μg/ml Lpp1, applied from the basolateral side. *F*, TEER readings of Caco2 BBE monolayer in 96-well-plate before and after the treatment with different combinations of 0.1 μg/ml InvE, 0.1 μg/ml sipC, or 0.5 μg/ml Lpp1, applied apically. Note, the medium control for (*E* and *F*) was used as a baseline for these individual treatments ran in the same experiments. *G*, TNFα ELISA was performed on Caco2 BBE cells treated with blank medium, PBS, Lpp1 (0.5 or 1 μg/ml), InvE (0.5 μg/ml), sipC (0.1 or 0.5 μg/ml), and YbhG (5 μg/ml). *H*, cell death was determined by Trypan blue analysis on cells with the above treatments. *I*, immunofluorescent staining for ZO-1 on Caco2 BBE cells with the above treatments. Arrows point to the perturbed junctional structures by sipC, InvE, and Lpp1. ∗*p* < 0.05, ∗∗*p* < 0.01, ∗∗∗*p* < 0.001, ∗∗∗∗*p* < 0.0001. The length of scale bars in (*I*) is 50 μm.

InvE, at a concentration of 0.5 μg/ml or above significantly decreased TEER 1 day after treatment. While cells treated with 0.5 μg/ml InvE sustained their TEER for up to 7 days, higher InvE concentrations abolished TEER entirely (Fig. 7*A*). In contrast, cells treated with 0.1 μg/ml did not show any change in the TEER (Fig. 7*A*). sipC treatment significantly reduced TEER reading even at a concentration of 0.1 μg/ml. Higher concentrations of sipC diminished TEER on the first day of treatment (Fig. 7*B*). Likewise, cells treated with 1 or 5 μg/ml of Lpp1 demonstrated a reduced TEER, while lower concentrations did not affect TEER (Fig. 7*C*). Treating cells with YbhG (UPF 0194), an uncharacterized membrane protein, did not elicit any TEER reduction (Fig. 7*D*). Interestingly, when InvE and Lpp1 were applied from the basolateral side of the monolayers, no significant TEER reduction was detected, whereas 0.5 μg/ml sipC induced a slight TEER reduction on days 2 and 3 from the basolateral side (Fig. 7*E*).

Since 0.1 μg/ml InvE, 0.1 μg/ml sipC, and 0.5 μg/ml Lpp1 did not induce a pronounced TEER reduction, we investigated their potential synergy in perturbing the epithelial barrier. We evaluated all potential combinatory effects of any two or all three of these factors of the above concentrations. A clear synergistic effect was observed on sipC and Lpp1 combination (green, Fig. 7*F*), and the triple combination elicited the most significant TEER reduction (purple, Fig. 7*F*).

Lpp1-induced TNFα production as an immune response (36, 40). TNFα is proinflammatory and can impair epithelial and tight junction integrity (41, 42, 43). To test of any of the above proteins may elicit TNFα production from Caco2 BBE monolayers, we treated cells with individual proteins at a non-lethal concentration, for 24 h and measured TNFα by ELISA. We found that 1 μg/ml Lpp1 elicited 8-fold above the baseline, and the highest production of TNF-a (*p* < 0.0001) (Fig. 7*G*). With 0.5 μg/ml InvE and 5 μg/ml YbhG treatments, no change was observed (Fig. 7*G*). However, 0.1 μg/ml of sipC slightly increased TNFα (*p* = 0.0007), and this induction appeared to be sipC dose-dependent (Fig. 7*G*). YbhG does not affect TNFα production. Based on trypan blue analysis, we found that low dosages of InvE and sipC treatments caused some cell deaths after 24 h, while Lpp1 treatment had minimal impact on cell death (Fig. 7*H*). With the same treatment as the Trypan Blue test, we stained ZO-1 on the Caco2 BBE cell. Interestingly, InvE, sipC, and Lpp1 seem to drastically perturb the epithelial cell tight junction structures, morphology, and localization of ZO-1 based on confocal immunofluorescent analysis (Fig. 7*I*).

## Discussion

Lysozyme was reported to have bactericidal activity on both Gram-positive and Gram-negative species (4, 44, 45, 46), while it displays a stronger bactericidal activity towards Gram-positive ones *in vitro* (4). *S. Typhimurium* is Gram-negative, and with the experiments performed by us, we failed to detect any bactericidal effects of human or chicken lysozymes on the growth or invasion by *Salmonella* under aerobic or anaerobic conditions. In our previous study, we found that elevated levels of intestinal lysozyme exacerbate chemically-induced inflammation whereas deleting Lyz1 protects from the inflammatory responses (6). Here, we demonstrated that during *Salmonella* infection, lysozyme overexpression increased bacterial invasion and the inflammatory response, whereas Lyz1 deficiency significantly protected against pathogen-induced inflammatory response. These findings are in agreement that intestinal lysozyme may play a pro-inflammatory role during disease progression.

Because lysozyme shapes the intestinal bacterial landscape under a steady state. Specifically, *Villin-Lyz1*^*TG*^ mice have increased Bacteroidetes and decreased Firmicutes compared to wild-type mice (6). We suspected that this changed microbial composition may contribute to *Salmonella* susceptibility. Thus, we used antibiotics to pretreat mice before infection and observed a similar barrier disruption in *Villin-Lyz1*^*TG*^ mice as well as protection in *Lyz1*^*−/−*^ mice following infection. These data suggest that a direct interaction between lysozyme and *Salmonella* may contribute to disease exacerbation.

Our data suggest that lysozyme does not exhibit a bactericidal effect against *Salmonella*, reflected as either the growth or invasion *in vitro*. However, the supernatant from lysozyme-treated *Salmonella* cultures damaged epithelial barrier function in the human Caco2 cells or in enteroids. These data are consistent with an increased *Salmonella* invasion in *Villin-Lyz1*^*TG*^.

*Salmonella* invades epithelial cells by actively secreting virulent factors through the T3SS (18, 47). Through proteomic analysis, we identified significantly increased releases of *Salmonella*-derived factors that were stimulated by the direct engagement with lysozyme. These factors include components of the T3SS, *i.e.*, InvE and sipC, as well as major outer membrane protein Lpp1 (36), all of which may drive the intracellular invasion by the pathogen *via* multiple parallel pathways (36, 48, 49).

Interestingly, InvE is a T3SS cell invasion protein also found in other bacteria species, such as *Escherichia coli* (48, 50, 51, 52, 53), suggesting that the observed lysozyme-stimulated InvE production may also apply to non-*Salmonella* species. T3SS is a transmembrane apparatus utilized by *Salmonella* and other invasive pathogens to engage with the host cell membrane, assemble translocon then inject effector proteins, including SopB, SopE, sipB, sipC, InvA, and InvE (18, 19, 20). Without activation of the T3SS, InvE is not typically released. Our data suggest that lysozyme’s engagement with *Salmonella* may provoke an aberrant activation of the T3SS. As InvE recruits sipB, sipC, sipD and sscA to form a translocon complex on the host cell membrane (19), we indeed also detected an elevated sipC abundance in lysozyme-treated cultures or in infected *Villin-Lyz1*^*TG*^ mice with a modified gain-of-function in lysozyme. sipC is known to hijack host cellular F-actin and CDC42 to facilitate bacterial invasion, and previous studies demonstrated an interaction of InvE with F-actin (54, 55). These reports are consistent with our findings that InvE and sipC drastically perturbed the epithelial junctional morphology and TEER in Caco2 monolayers.

Lpp1 is a *Salmonella* major outer membrane protein, a deficiency of which decreases its cellular invasion (36, 40). Lpp1 has been shown to induce host cells to produce TNFα (40) as also observed in our experiments. However, to our knowledge, we demonstrated for the first time that Lpp1 directly impairs the epithelial barrier and increases gut permeability in Caco2 monolayers. Although a low concentration of TNFα helps epithelial wound healing (56) *via* the Wnt pathway (57, 58), a high concentration of TNFα can induce cell death, damage the epithelial integrity through MLCK activation (41, 42), perturb barrier function *via* Occludin endocytosis (43), and drive pathogen invasion (59, 60, 61). It is plausible that enhanced Lpp1 release triggered by lysozyme-*Salmonella* interaction provokes a feed-forward mechanism, indirectly mediated through TNFα for barrier disruption and *Salmonella* invasion. This effect could be appreciated from the observed temporal TEER changes elicited by Lpp1 in our time-course experiments. InvE, sipC, and Lpp1 all induced ZO-1 perturbation and TEER reduction. Thus, when the paracellular pores increase to 62 Å radius due to ZO-1 perturbation (43), *Salmonella* and other Gram-negative bacteria, such as *E. coli*, can cross the epithelial barriers. These changes may be reflected by the contrasting phenotypes observed in lysozyme-sufficient *versus* deficient mouse models. Thus, we propose that lysozyme engagement with *Salmonella* drives the release of virulence factors jointly perturbed epithelial tight junction integrity *via* T3SS- and TNFα-mediated mechanisms.

Interestingly, *Bacteroidetes* are anaerobic Gram-negative rods that employ the Type IX Secretion System (T9SS) to release virulence proteins (62, 63, 64, 65). Both T3SS and T9SS play essential roles in transferring virulence factors from the bacterial matrix to host cells or the intercellular space. *Bacteroidetes* are increased by approximately 20% in Villin-Lyz1^TG^ mice (6), therefore lysozyme may use a similar mechanism on *Bacteroidetes* to release virulence factors controlled by T9SS. Indeed, we observed an increased intestinal permeability in *Villin-Lyz1*^*TG*^ mice under homeostatic conditions compared to WT mice. This line of investigation should be pursued in the future.

Our study is clinically relevant as abnormal production of human c-type lysozyme was reported in various intestinal inflammatory diseases (7, 8, 9, 10, 11, 66, 67, 68, 69). While no causation has been established to link any single agent to chronic IBD, enteric pathogens and opportunistic pathobionts are associated with the development or exacerbation of gut inflammation (70, 71, 72). *S. Typhimurium*, *Campylobacter* (73), *E. coli* (74), *Helicobacter pylori* (75), and *C. difficile* (76) among others, have been linked to intestinal inflammation. Population cohort studies showed that individuals with gastrointestinal infection by *Salmonella*/and *Campylobacter* before disease onset had an increased IBD risk (73). A portion of later-diagnosed patients with IBD presented serum antibodies against several bacterial and fungal antigens (77), and the disease progression and aggravation were directly correlated with serum toxins of *Salmonella* and other enteric pathogens (78), anti-*Salmonella* antibodies (79), and antibodies against *Saccharomyces cerevisiae*, *E. coli, P. fluorescens* (80). While some studies suggested that microbial dysbiosis driven by the course of IBD may increase the risk of infection by pathogen (81, 82), others analyzing antibiotics therapy data for IBD standard care attributed the disease causative to pathogenic agents (83). By reporting a potentially deleterious role of host lysozyme in promoting inflammatory progression during infection, our study points to the potentially context-dependent mechanism underlying the host-pathogen interaction. The observed reduction of lysozyme expression in Paneth cells in response to *Salmonella* infection shown by us and others (17) may reflect an adaptive protection by the host. It would be interesting to delineate the mechanism behind the pathogen-activated circuit responsible for activating this host protection.

We did not observe barrier-regulating effects by YbhG, which is associated with the survivability of bacteria (37, 38). Also, we did not test all the other possible targets such as the SPI-6-associated protein, the hypothetical membrane proteins, or chaperones, primarily due to a lack of available reagents for these proteins. Our data suggest that the lysozyme-induced virulence factor release depends on lysozyme’s enzymatic function, as heat-inactivated lysozyme failed to elicit TEER response. A previous study reported that heat-denatured lysozyme still maintained bactericidal function (84). confirmed that the pliC inhibits the barrier-disrupting effects resulting from lysozyme-mediated *Salmonella* processing. pliC is known to bind the bactericidal domain H-35 while also blocking lysozyme’s enzymatic function (85). Future work will also determine how lysozyme promotes *Salmonella* to release these factors at the molecular level.

## Experimental procedures

### Bacterial strain, culture, and growth analysis

*Salmonella enterica Typhimurium* strain (ATCC SL1344 or ATCC14028) (86) was cultured in Luria-Bertani (LB) broth (Sigma L3022) with 100 μg/ml ampicillin (Sigma A9518-25G) at 37 °C with shaking.

Bacterial growth was measured at OD600 by a NanoDrop ONE^C^ (Thermo Scientific), with cell numbers determined based on OD600 of 1.0 = 8 × 10^8^ cells/ml. Aerobic culture condition was used for animal inoculation studies. Anaerobic culture was done in an anaerobic chamber (PLAS LABS 857-OTA) only for testing lysozyme’s effects and was specified in the experimental results. Growth curves were plotted in GraphPad Prism 10, with OD600 values determined hourly after different treatments.

### Caco2 BBE cell culture, TEER, and FITC-dextran flux measurement

The Caco2 BBE cell line was cultured in DMEM supplemented with 20% fetal bovine serum (FBS) 1% Pen-strep (Thermo Fisher, 15140-122), and 0.2% Primocin (Thermo Fisher, NC9141815). The cells were grown at 37 °C with 5% CO_2_. The culture medium was replenished every 2 days. Cells were passaged at 80% confluence. For TEER analysis, 2 × 10^5^ Caco2 BBE cells were initially seeded in 96-well 0.4 μm pore size Transwell plate (STEMCELL, 100-0419). For FITC-dextran permeability cells were seeded in 6-well 0.4 μm pore size Transwell inserts (Sigma Millipore, CLS3450). After a minimum of 16 days, cells were grown into a differentiated monolayer when the TEER reading is stabilized for 3 consecutive days. Treatments were applied to the monolayer from either apical or basolateral side specified in the experimental results. Apical chamber holds 70 μl medium with different concentrations of treatment, while basolateral compartment contains 250 μl medium. Every day, cells were washed with PBS and fresh medium was changed in both chambers. TEER readings were then collected by EVOM (World Precision Instruments, EVM-MT-03-01) at a consistent time of a day. For FITC-dextran flux measurement, medium was changed to FluoroBrite DMEM without phenol red (Thermo Fisher, A1896701). Apical medium containing 10 mg/ml 4 kDa FITC-dextran (Sigma, FD4) was supplemented with 5 or 10% of filtered-sterilized LB broth from (i) 16 h *Salmonella* culture without lysozyme; (ii) LB broth with 4,000U/ml of human lysozyme without bacteria; (iii) conditioned medium from *Salmonella* culture treated with 4,000U/ml of human lysozyme for 16 h.

### Mice

*Lyz1−/−*, *Villin-Lyz1*^*TG*^, and *Lyz1*^*3’UTR-IRES-CreER*^*; Rosa26R-tdTomato* mice have been described previously (6, 23, 87). All mice are maintained on C57BL/6 background. Experimental procedures are approved by the Rutgers University Institutional Animal Care and Use Committee. Mice are kept on a 12-h light and dark cycle, given unlimited access to food and drink, and kept in separately ventilated cages free of specific pathogens. All tests were carried out on littermates that were genetically altered or wild type, of both sexes. Power analysis was used to determine the animal numbers based on intra-group experimental variabilities. Mouse numbers were reflected in each data plot.

### *Salmonella* virulence factors

Lpp1 (catalog no. CSB-EP745252SXB) (36), YbhG (CSB-EP856117SXB) (88), and InvE (CSB-EP339573SXB) (48) were purchased from CUSABIO TECHNOLOGY LLC. These proteins were 6xHis-tagged at C-terminal, expressed in *E. Coli*, purified, and lyophilized in 20 mM TRIS-HCl, 0.5 M NaCl, 6%Trehalose, pH 8.0. The purity of Lpp1 and YbhG is 93%, and InvE is 89%. sipC (catalog. MBS1016763) (89) and pliC (MBS 1177063) (https://www.uniprot.org/uniprotkb/P0AD59/entry) were purchased from MyBioSource LLC. These proteins were 6xHis-tagged at C-terminal, expressed in *E. Coli*, purified, and lyophilized from a filtered 20 mM Tris-HCl, 0.5 M NaCl, 6% Trehalose, pH 8.0. The purity of pliC is 93% and sipC is 85%. The quality of all these factors was examined by SDS-PAGE with 5% enrichment gel and 15% separation gel. These lyophilized proteins were reconstituted in PBS and filtered through a 0.22 μm filter, stored at −80 °C, used at a concentration described in the experiments.

### *Salmonella Typhimurium* infection

A single colony of *S. Typhimurium* SL1344 was inoculated in LB-ampicillin broth to allow growth for 16 h at 37 °C with shaking. 100 μl of the culture was inoculated to 5 ml of fresh LB-ampicillin broth, and cultured at 37 °C for 4 h 10^8^ or 10^9^ CFU *S. Typhimurium* (based on OD600) was inoculated to each mouse. Experiments involving antibiotic pretreatment was specified in the experimental results. Mouse body weight was recorded before and after bacterial inoculation daily. The mice were sacrificed 4 days after infection. Liver, spleen and MLN were used for *Salmonella* CFU determination as described previously (23). Briefly, tissues were weighed and homogenized by Misonix Sonicator (Qsonica LLC. XL-2000) in PBS at a ratio of 1 mg tissue per 10 μl PBS. Tissue lysates were plated on XLD Agar plate (Sigma Aldrich, 95586) with 10-fold serial dilutions and grown for 24 h in a 37 °C incubator at room temperature in the hood. Numbers of single colonies were counted in each region of different dilutions and averaged to obtain the CFU value per mg tissue. Results were plotted in GraphPad Prism 10 with CFUs transformed on a log_10_ scale.

### *In vivo* intestinal permeability assay

Four hours prior to sacrifice, food was removed and mice were gavaged with 60 mg/100g body weight fluorescein isothiocyanate–dextran (FITC-Dextran) (Sigma, FD4). Upon sacrifice, blood was removed aseptically by cardiac puncture in 5 mM EDTA and centrifuged at 3000*g* for 5 min. Plasma was separated from blood cell pellet for each sample. For quantification of FITC-Dextran in plasma samples, a standard curve was created through serial dilutions from 0.1 pg/ml to 1 ng/ml in control plasma. 100 μl of each standard and each experimental sample were transferred to a black-opaque-bottomed 96-well plate. Promega GloMax Plate Reader was used to determine fluorescence in samples with an excitation at 485 nm and emission at 530 nm.

### Tissue collection, immunofluorescence, and immunohistochemistry

Mouse intestinal tissues (ileum, colon, and cecum) were collected, fixed in 10% formalin overnight, then transferred into 70% ethanol and stored at 4 °C. Tissues were subjected to paraffin embedding, and 5 μm sections were cut from paraffin blocks, rehydrated, and subjected to H&E staining (Hematoxylin, Sigma-Aldrich, GHS116; Eosin, Sigma-Aldrich, HT110316). Alcian blue staining and procedures of immunofluorescence and immunohistochemistry were described previously (6, 23, 87). For immunostaining analysis, slides were treated in sub-boiling antigen retrieval buffer (1 μM citric acid, pH 6.0 or 1 μM EDTA, pH 8.0) for 10 min and then immediately transferred into running water. Slides were blocked with PBS buffer containing 0.1% Triton X-100, 2% Bovine Serum Albumin (Sigma A3294-100G), and 2% normal goat serum for 2 h at room temperature, and then incubated with indicated primary antibodies at 4 °C overnight. The next day, the slides were washed with PBS, and incubated with biotinylated or immunofluorescent secondary antibodies for 1 h at room temperature. Immunohistochemistry was developed by using the ABC kit (Vector Lab, PK-4000), DAB kit (Vector Lab, SK-4100), and imaged by Nikon TE 2000D with NIS Elements D version 4.4. Fluorescent images were taken by Zeiss LSM 980, and analyzed by NIH Image J software.

### RNA BaseScope analysis

The RNA BaseScope *in situ* hybridization assays were performed following the manufacturer's instructions (Advanced Cell Diagnostics, ACD). Formalin-fixed paraffin-embedded tissue sections (5 μm) were stained by probe BA-Mm-Ang4-1zz-st targeting the mouse *Ang4*, and probe Ba-Mm-Lyz1-3zz-st targeting the mouse *Lyz1*. mRNA was detected by BaseScope Duplex Detection Reagent Kit, which was previously described (23).

### ELISA assay

Tissues or Caco2 cells were lyzed by 50 mM Tris (pH7.5), 150 mM NaCl, 10 mM EDTA, 0.02%NaN3, 50 mM NaF, 1 mM Na3VO4, 0.5%NP40, 1 mM PMSF, 0.5 mM DTT and protease inhibitors (Sigma), homogenized by sonication, and centrifuged to collect supernatant. Concentrations of protein targets were determined by Protein Assay Dye Reagen Concentrate (Bio-RAD #5000006). 20 μg of total protein was used in each sample. Lipocalin 2 (LCN2) was measured by Quantikine ELISA Mouse Lipocalin-2/NGAL Immunoassay Kit (R&D System, Catlog# MLCN20) following manufacture’s procedures. For TNFα ELISA, mouse TNFα DuoSet (R&D DY410-05) was used. For sipC ELISA, anti-sipC IgG (TGC Biomics, tgc-A203-1) was used as capture antibody and HRP-conjugated anti-Rabbit antibody (Invitrogen, NA934V) was used as detection antibody. The results were read at a wave length of 540 nm by Promega BioSystems Glomax Multi.

### FITC-dextran microinjection in enteroids

Human enteroids were passaged and resuspended fragments were mixed with fresh Matrigel at an appropriate ratio to achieve the desired enteroid density. The mixture was spread onto pre-warmed 4-well coverslip chambers. After solidifying at 37 °C for 30 min, the enteroids were overlaid with complete enteroid growth medium. The enteroids were grown for 5 days before injections were performed. Using microinjection hardware, organoids were treated with 59 nl of FITC-dextran diluted in PBS at a concentration of 5 ug/ul. The treated organoids were subsequently imaged using Nikon microscope to assess luminal FITC intensity. The organoids were then treated with the respective media containing microbial toxins or vehicle (PBS). After 24 h luminal intensity was captured using the same settings used on the previous day to quantify loss of luminal FITC intensity.

### Computational analysis of Paneth cell scRNA-seq data

The count matrices of scRNA seq are from S. Typhimurium-infected (SAL) and uninfected (PBS) Paneth cell data from previous publications (23). The Paneth cell scRNA dataset is available in Gene Expression Omnibus (GEO) with an accession number: GSE237326. Briefly, Lyz1^3'UTR-IRES-CreER^; Rosa26R^tdTomato^ Paneth cell reporter mice were intraperitoneally injected with tamoxifen to activate the tdTomato in Paneth cells, 24 h before PBS or *Salmonella* gavage. After 4 days of *Salmonella* infection, Paneth cells were isolated from ilea of uninfected or infected mice, and were used for single-cell RNA sequencing analysis by 10 × Genomics. The matrices had been loaded into R (version 4.3.1) and were combined and used as the input for the Seurat package (version 4.4.0) (90). Cells with a high percentage of total unique molecular identifier (UMI) counts originating from mitochondrial RNA and cells with low UMI counts were filtered out. Clustering analysis was performed using the FindClusters function with a resolution parameter of 0.8. The clusters were visualized using the Uniform Manifold Approximation and Projection (UMAP) algorithm (91) with principal components as input and dims = 30, n.neighbors = 30. The clusters had been annotated as Paneth cell progenitors and mature Paneth cells based on the expression levels of stem cell markers (Lgr5, Olfm4, and Stmn1) and Paneth cell markers (Reg3g, Mptx2, and Lyz1).

### Proteomics for lysozyme-processed *Salmonella*

*S. enterica Typhimurium* (ATCC SL1344) was grown on LB agarose (35 g/1L DD water) (Sigma L2897) at 37 °C for 48 h. A single bacteria colony was picked and inoculated in 25 ml autoclaved LB broth (Sigma L3022) with 25 μl ampicillin (Sigma A9518-25G) for 16 h at 37 °C. 100 μl was transferred to fresh LB broth and inoculated for another 4 h. Cells were centrifuged at 3500 rcf for 10 min. The medium was removed, and cells were resuspended in PBS and measured at OD600 by NanoDrop ONE^C^ (Thermo Scientific). 10^9^ cells were suspended in PBS with or without 0.2 μg/ml Human lysozyme (Sigma L1667). Reaction was incubated at 37 °C for 24 h. Samples were centrifuged at 3500 rcf for 10 min. Supernatant was collected, and protein concentrations were determined by Bradford assay. Two mg of total proteins per sample were denatured in LDS and resolved by SDS–PAGE (Invitrogen NP0335BOX). Coomassie Blue staining was performed and protein bands were cut and subjected to in-gel digestion and mass spectrometry.

### *In vitro Salmonella* invasion assay

Caco2 BBE was seeded at 106 cells/well in 6-well plates (35 mm). Cells were grown to differentiated monolayers and then maintained for 2 weeks. Cell culture media was replaced with 20% Fetal Bovine Serum (FBS) in DMEM. Antimicrobial peptides were added 15 min at indicated concentrations prior to addition of bacteria. Antimicrobial peptides we used were 0.175 μg/ml SAP (R&D, 1948-SAB), 0.23 μg/ml CRP (R&D, 1707-CR-200), 0.5 μg/ml Reg3b (R&D, 5110-RG-050), 0.05 μg/ml Retnlb (Abnova, P4630), 0.2 μg/ml α-defensin (Novus Biologicals, H00001670-P01), 0.5 μg/ml β-defesin (Thermofisher, PHC1624), 0.2 μg/ml human lysozyme (Sigma, L1667-1G), 0.05 μg/ml Ang4 (R&D 964-AN-025) and 150 μg/ml. SL1344 was added to cells at a MOI of 100 and incubated with cells for various time points (up to 30 min) at 37 °C, 5% CO2. Cells were then washed with 150 μg/ml gentamicin (Corning, #30-005-CR) for 50 min after removal of bacteria, washed once with PBS, lysed with 0.5% Triton X-100 in PBS, and serial dilutions of lysates were plated on XLD agar plates for quantifying bacterial growth.

### Trypan blue staining

Caco2 BBE was seeded at 10^4^ cells/chamber in 8-chamber slide. Cells were grown to differentiated monolayers and then maintained for 3 days. Cell culture media was replaced with 20% Fetal Bovine Serum (FBS) in DMEM. 0.5 μg/ml of InvE, 0.1 μg/ml of sipC, and 1 μg/ml of Lpp1 were treated in the chambers. Medium and PBS were used as control and vehicle. Within 24 h of treatment, cells were stained with 1:10 diluted Trypan blue (Invitrogen, T10282). Cell viability was determined by counting both stained (non-viable) and unstained (viable) cells. The percentage of viable cells was calculated by dividing the number of unstained cells by the total number of cells counted.

### Quantification and statistical analysis

All statistical analysis was done using the GraphPad Prism software unpaired t-tests for 2-group comparison, and two-way ANOVA test for multigroup analysis. Each experiment contained 3 to 11 mice per group. Quantification of immunostaining results was reported from 6 to 10 independent microscopic fields from at least 3 mice for each condition. Mouse numbers per group are reported in individual figure legends. Area and intensity in immunofluorescence staining were determined by NIH Image J. Multi-channel images were split into individual channels using the split channel function. A region of interest was set, then area and intensity functions were determined for channels of interest.

## Data availability

Data, analytic methods and study materials will be made available to other researchers upon written request. Mass spectrometry proteomics data have been deposited to the ProteomeXchange Consortium *via* the PRIDE partner repository with the dataset identifier PXD049696.

## Conflict of interest

The authors declare that they have no known competing financial interests or personal relationships that could have appeared to influence the work reported in this paper.
